# Supplementary material for: Electronically decoupled stacking fault tetrahedra embedded in Au(111) films
Source: Nat Commun. 2016 Dec 23;7:14001. doi: 10.1038/ncomms14001 (PMC5196436; doi:10.1038/ncomms14001)
Supplement: Supplementary Information — Supplementary Figures and Supplementary References [file ncomms14001-s1.pdf]

Supplementary Figures

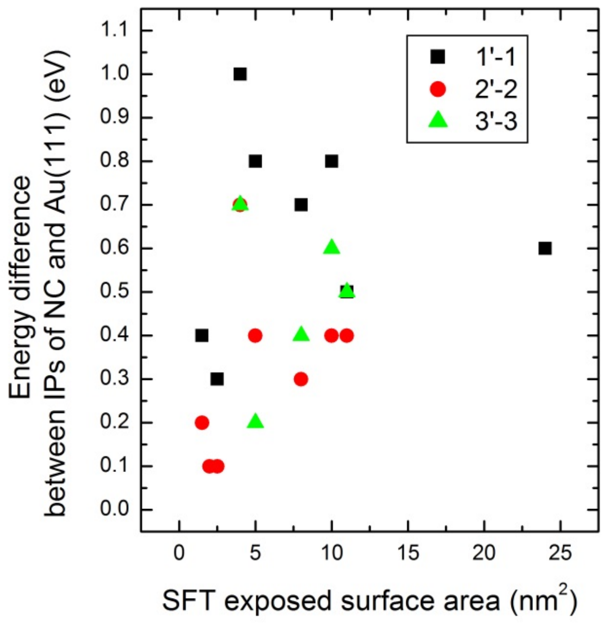

Supplementary Figure 1 │ Energy differences between the image-potential states Energy (voltage) difference between the three lowest image-potential states recorded above Au stacking fault tetrahedra (SFTs) (labelled n = 1’, 2’, and 3’ in Fig. 2d) of various size and the corresponding image-potential states recorded on the surrounding Au(111) surface (labelled n = 1, 2, and 3 in Fig. 2d). There apparently does not exist a direct relation between the voltage differences and the SFT size. The average difference for states 1’-1, 2’-2, and 3’-3 is 0.6 ± 0.2 eV, 0.3 ± 0.2 eV, and 0.5 ± 0.2 eV, respectively. The scatter of the data can be at least partially ascribed to (uncontrolled) changes of the scanning tunnelling microscopy (STM) tip apex during the (often long-time) search for different SFTs across the Au(111) surface. The STM tip apex determines the effective electric field between tip and sample and hence the Stark shift of the states. Moreover, due to the increased tip-sample distance (and hence decreased spatial resolution) in image-potential state measurements, the tunnelling current recorded above the Au SFTs will also include a tunnelling contribution of the surrounding Au(111) surface (in particular for the smallest Au SFTs), which can further influence the determination of the image-potential states of the Au SFTs.

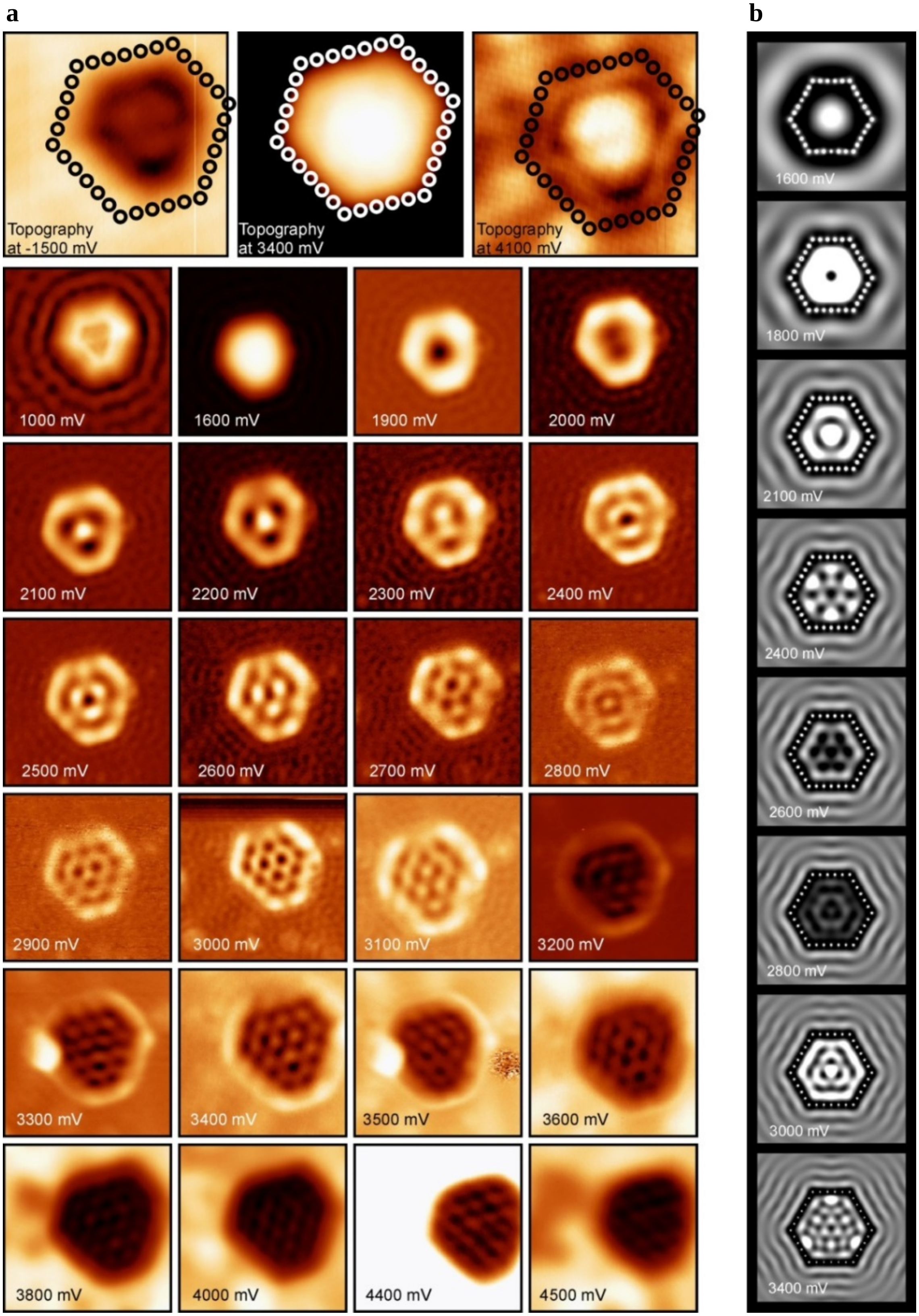

Supplementary Figure 2 | Comparison of the experimental maps to the particle-in-a-box model Panel a presents three topography images (image size: 7 x 7 nm^2) and an extended series

of obtained dI/dV maps of the Au stacking fault tetrahedron presented in Fig. 3, recorded at the indicated voltages. The images in panel b present simulated local density of states images following the 2D particle-in-a-box model using the confining geometry (consisting of adatom scattering centers) indicated in the scanning tunnelling microscopy topography images and using E_0 = 1490 meV and m^* = 0.33 m_e. Simulations are performed using the "particle-in-a-box" software (available via Ref. [1]) developed by K.-F. Braun[2].

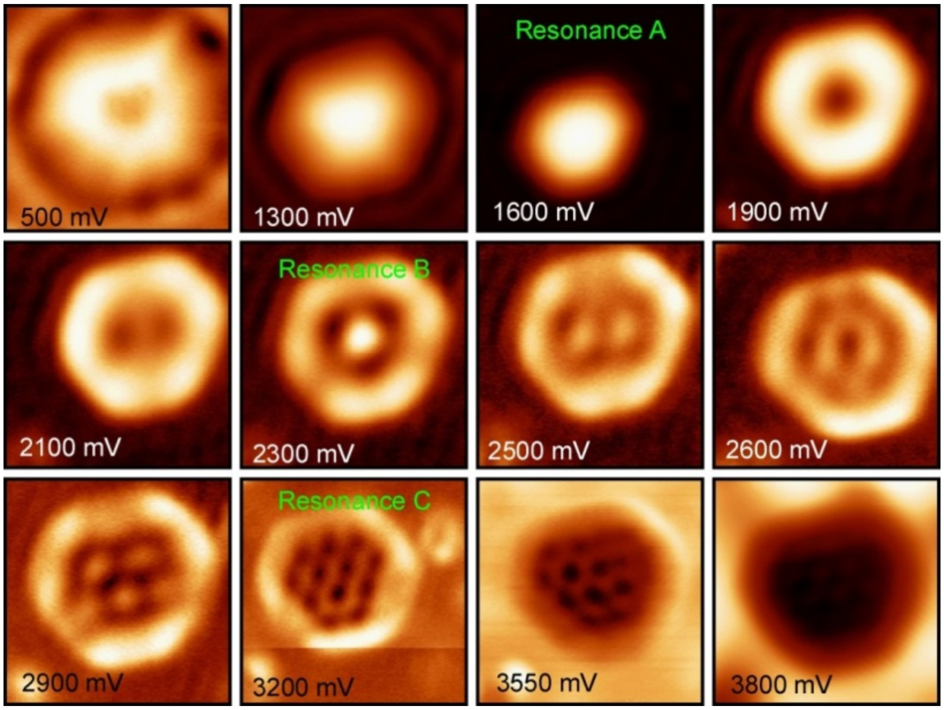

Supplementary Figure 3 | Experimental maps of the Au stacking fault tetrahedron (SFT) in Figs. 2a and 2b Series of dI/dV maps (closed feedback loop) of the Au SFT presented in Figs. 2a and 2b, recorded at the indicated voltages. Image sizes are 6 x 6 nm^2. Corresponding dz/dV spectra, which are recorded in the center of the SFT, are presented in Fig. 2c. dI/dV maps that show a local maximum in the SFT (i.e., at 1600 mV, 2300 mV, and 3200 mV) center reflect the eigenstates corresponding to the resonance maxima labelled A, B, and C in Fig. 2c. At voltages above the bottom of the Au(111) bulk conduction band, the wave patterns within the Au SFT surface become difficult to discern due to the increased density of states of the surrounding Au(111) surface.

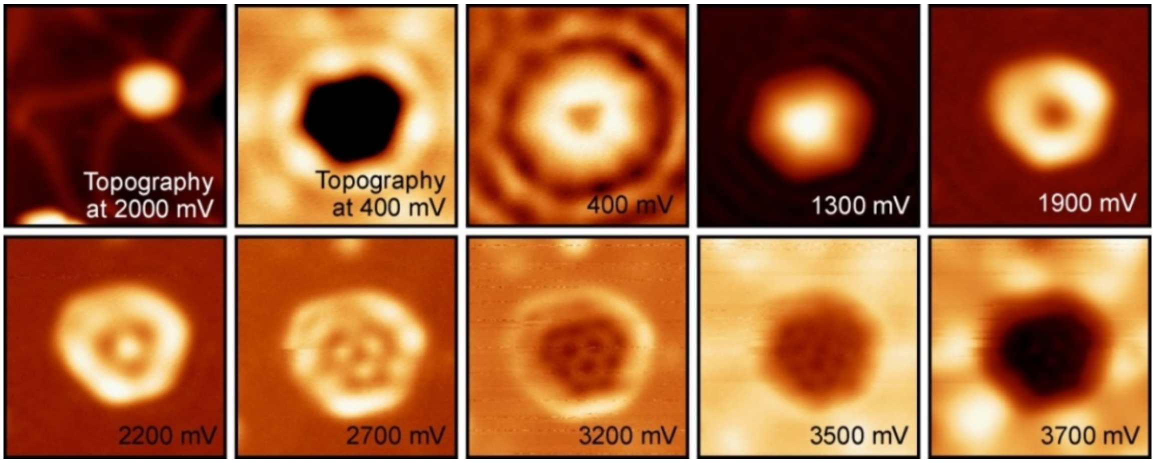

Supplementary Figure 4 │ Experimental maps of another stacking fault tetrahedron (SFT) Topographies (V = 2000 mV with image size of 15 x 15 nm^2; V = 400 mV with image size of 8 x 8 nm^2) and corresponding series of dI/dV maps (closed feedback loop; image sizes are 8 x 8 nm^2) of another Au SFT, recorded at the indicated voltages. The Au SFT has very similar size and shape as that in Supplementary Fig. 4, and shows quasi-identical wave patterns and voltage-dependent behaviour (compare, e.g., maps at 1300 mV, 1900 mV and 3200 mV).

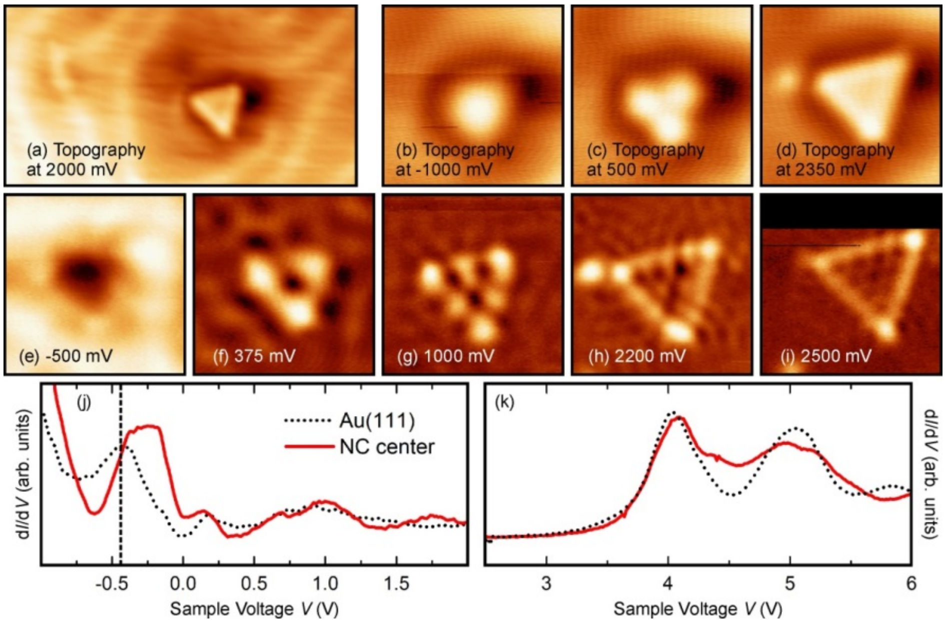

Supplementary Figure 5 │ Experimental maps and spectroscopy data of a triangular defect-like feature (a) Topography (*V* = 2000 mV; image size is 23 x 12 nm^2) of an exceptionally observed triangular defect-like feature (only one observation). The surrounding herringbone ridges have an organization that differs from that of the other here reported stacking fault tetrahedra (SFTs). (b)-(d) Corresponding close-up view topographies at the indicated voltages. Image sizes are 7 x 7 nm^2. (e)-(i) Corresponding series of d*I*/d*V* maps (closed feedback loop; image sizes are 7 x 7 nm^2), recorded at the indicated voltages. (j) d*I*/d*V* spectra (open feedback loop; *V* = 2.0 V, *I* = 1.0 nA) reveal a resonance at the defect center that is shifted to higher voltages compared to the onset of the Au(111) surface state at –460 meV (indicated by dashed vertical line). This resonance and the wave patterns in (b)-(d) can be attributed to confinement of the bare Au(111) surface state by the stacking faults. (k) d*I*/d*V* spectra (open feedback loop; *V* = 2.0 V, *I* = 1.0 nA) reveal the onset of the bulk conduction band of the Au(111) surface also on the defect, which is not the case for the other here reported Au SFTs. A possible conclusion is that the triangular particle is a two-dimensional defect rather than a three-dimensional defect such as the other here reported Au SFTs. In particular, the triangular feature may be interpreted as a so-called Frank loop, consisting of a single stacking fault. The Frank loop is very similar to the SFT in terms of the spatial coordinates of atoms.

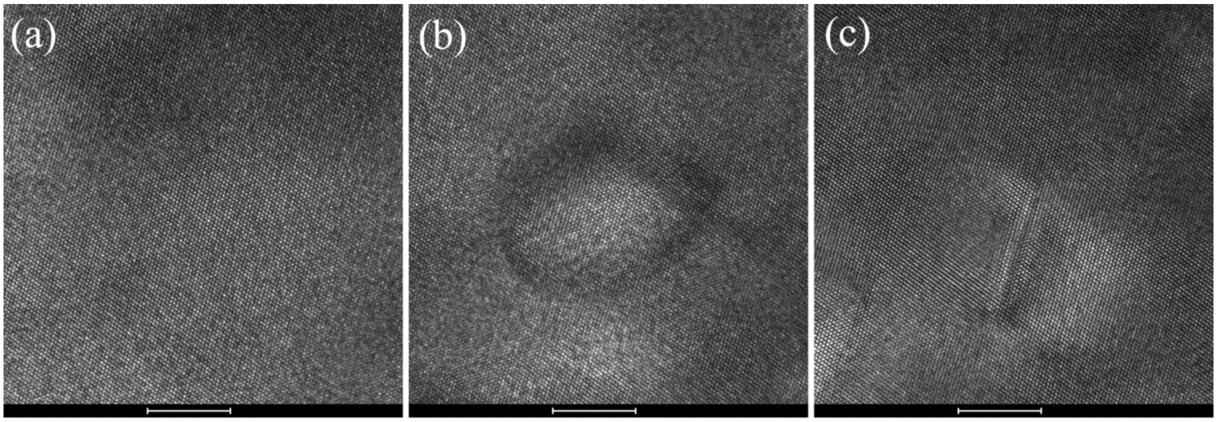

Supplementary Figure 6 | High-resolution (HRTEM) data of a Au reference sample HRTEM investigation along <110> direction of a cross-sectional Au focused ion beam (FIB) sample used as reference. Prior to the FIB treatment, the bulk Au was annealed at 973 K for 24 h in order to remove all 1D and 2D defects. In (a) no irradiation damage can be observed, in (b) the signature of a dislocation loop can be recognized and in (c) a single stacking fault with strain field is seen. However, over the entire size of the FIB sample (approximately 3 by 3 micron^2) no signature of a stacking fault tetrahedron (SFT), similar to the SFTs found in the Au films on mica, was observed. This indicates that the used FIB thinning parameters (see Methods section in the main text) do not result in the creation of SFTs in Au. The scale bars in (a)-(c) correspond to a length of 5 nm.

Supplementary References

(1) Braun, K.-F. Calculation of standing wave pattern and image processing. Available online at http://www.kai-felix-braun.de/program.htm (2006).

(2) Braun, K.-F. & Rieder, K.-H. Engineering electronic lifetimes in artificial atomic structures. *Phys. Rev. Lett.* **88**, 096801 (2002).
